# Novel ethanol production using biomass preprocessing to increase ethanol yield and reduce overall costs

**DOI:** 10.1186/s13068-020-01839-0

**Published:** 2021-01-07

**Authors:** Danielle Uchimura Pascoli, Azra Suko, Rick Gustafson, Heidi L. Gough, Renata Bura

**Affiliations:** School of Environmental and Forest Sciences, University of Washington, Box 352100, Seattle, WA 98195-2100 USA

**Keywords:** Biomass preprocessing, Biomass wash, Whole-tree chips, Poplar, Ethanol yield, Overliming, Water recycling, Economic assessment

## Abstract

**Background:**

Ethanol biorefineries need to lower their overall production costs to become economically feasible. Two strategies to achieve this are to reduce costs using cheaper feedstocks or to increase the ethanol production yield. Low-cost feedstocks usually have high non-structural components (NSC) content; therefore, a new process is necessary to accommodate these feedstocks and overcome the negative effects of NSC. This study developed a novel ethanol biorefinery process including a biomass preprocessing step that enabled the use of lower-cost feedstocks while improving ethanol production without detoxification (overliming). Two types of poplar feedstocks were used, low-quality whole-tree chips (WTC) and high-quality clean pulp chips (CPC), to determine if the proposed process is effective while using feedstocks with different NSC contents.

**Results:**

Technical assessment showed that acidic preprocessing increased the monomeric sugar recovery of WTC from 73.2% (untreated) to 87.5% due to reduced buffering capacity of poplar, improved sugar solubilization during pretreatment, and better enzymatic hydrolysis conversion. Preprocessing alone significantly improved the fermentability of the liquid fraction from 1–2% to 49–56% for both feedstocks while overliming improved it to 45%. Consequently, it was proposed that preprocessing can substitute for the detoxification step. The economic assessment revealed that using poplar WTC via the new process increased annual ethanol production of 10.5 million liters when compared to using CPC via overliming (base case scenario). Also, savings in total operating costs were about $10 million per year when using cheaper poplar WTC instead of CPC, and using recycled water for preprocessing lowered its total operating costs by 45-fold.

**Conclusions:**

The novel process developed in this study was successful in increasing ethanol production while decreasing overall costs, thus facilitating the feasibility of lignocellulosic ethanol biorefineries. Key factors to achieving this outcome included substituting overliming by preprocessing, enabling the use of lower-quality feedstock, increasing monomeric sugar recovery and ethanol fermentation yield, and using recycled water for preprocessing. In addition, preprocessing enabled the implementation of an evaporator-combustor downstream design, resulting in a low-loading waste stream that can be treated in a wastewater treatment plant with a simple configuration.

## Background

Lignocellulosic ethanol is an attractive biofuel because it is renewable, has reduced environmental impacts, and avoids competition with the food industry. However, the high production cost of lignocellulosic ethanol makes this biofuel unable to compete economically with gasoline and first-generation ethanol. Gnansounou and Dauriat assessed the ethanol production costs using different feedstocks, such as straw, poplar, and switchgrass, and the authors concluded that, for all three cases, the feedstock is the biggest cost contributor to the total production cost (50–55%) [1]. Their sensitivity analysis also showed that a higher ethanol yield (i.e., liters of ethanol per ton of feedstock) results in lower feedstock usage per liter of ethanol, thus decreasing the feedstock costs. With this in mind, the present work presents a new biorefinery process focusing on two strategies to make lignocellulosic ethanol production economically feasible: (1) use a cheaper feedstock, which directly reduces the feedstock cost, and (2) increase the ethanol production yield, which results in both higher plant production capacity and lower feedstock cost relative to production rate.

In the Pacific Northwest, poplar is a potential feedstock candidate for biofuels production because of its high sugar content, all year round availability, fast growth rate, and minimum water and nutrient requirements [2]. A lower-cost option of poplar biomass is the short rotation coppice (SRC) poplar, which grows for only 2–3 years and has a whole-tree harvesting system that does not require debarking. SRC comprises a heterogeneous mixture of wood chips, bark, branches, and leaves [3]. Previous studies from our research group on bioconversion of SRC poplar found that leaves are very problematic due to their high ash and extractives content, and the removal of leaves was essential to achieve good bioconversion yields [3]. The leafless short-rotation poplar, called whole-tree chips (WTC) in this study, is a lower-cost wood feedstock of $77 per dry tonne (R. Stonex from GreenWood Resources, personal communication, March 2019). Historically, poplar trees have been used as raw material for the pulp and paper industry, where mature poplar trees are cleaned and debarked resulting in clean pulp chips (CPC) [3, 4]. More recently, this homogeneous biomass was also considered as feedstock for bioconversion purposes due to its superior quality (high sugar, and low ash and extractives content) [5]. However, the decade-long harvest cycles, labor-intensive harvesting, and extra cost for debarking the wood logs make CPC an expensive feedstock [3], costing on average $116 per dry tonne (R. Stonex from GreenWood Resources, personal communication, March 2019). Hence, even though CPC has superior quality relative to WTC, using the latter as feedstock for bioconversion may be preferable from the cost point of view.

Low-quality feedstocks such as agricultural residues, wood whole-tree chips, mixed biomass, among others, commonly have low sugar content and high ash and extractives (also called non-structural components) content. These non-structural components (NSC) are non-chemically bound components of lignocellulosic biomass that can directly affect pretreatment, enzymatic hydrolysis, and fermentation yields. Previous studies reported that ash increases the buffering capacity of the biomass, which negatively affects pretreatment efficiency [6–8]. Besides, Ranatunga et al. [9] reported that extractives from hardwoods can inhibit xylose fermentation by *Zymomonas mobilis* due to their antimicrobial properties, which directly reduces the ethanol yield. To overcome fermentation inhibition caused not only by extractives but from other compounds, such as sugar degradation products, different detoxification techniques are used to target specific types of inhibitors and improve the fermentation yield.

The National Renewable Energy Laboratory (NREL) developed a design for ethanol production from corn stover that used overliming detoxification to remove certain fermentation inhibitors and increase ethanol production [10]. Even though overliming is known to be effective in removing sugar degradation products and some types of phenols present in the liquid fraction after pretreatment by increasing the pH to high levels [11], it has been reported that overliming causes sugar loss of up to 20% due to side reactions at elevated pH, consequently compromising the final ethanol yield [12–14]. Another drawback of this technique is the formation of solid waste (gypsum) that requires proper separation and disposal, which increases the biorefinery’s operating costs [10]. For these reasons, NREL replaced overliming by whole-slurry ammonia conditioning, i.e., pH neutralization to ~ 5, in their 2011 lignocellulosic ethanol production design [15]. According to the report, ammonia conditioning is as effective as overliming while avoiding sugar loss and gypsum formation. However, the authors did not state the mechanism in which ammonia conditioning works and how it enhances fermentability without increasing the pH to higher levels. In addition, the replacement of overliming for ammonia conditioning resulted in a high concentration of ammonium salts in their stillage, which resulted in major changes in their design. NREL decided to remove the multiple-effect evaporator and send the stillage directly to waste treatment (WWT) instead, forcing them to completely change the WWT configuration to handle the high-loading waste stream. The installed equipment cost of their new WWT was $49.4 million, representing about 21% of the plant’s total installed cost [15]. For comparison, the previous NREL 2002 design that included overliming had a much simpler WWT design with an installed cost of only $3.3 million (representing 3% of the plant’s total installed equipment cost) [10]. Hence, both overliming and ammonia conditioning have their pros and cons, and the search for alternative methods with less problematic impacts is crucial.

Biomass preprocessing washing has been commonly used to remove ash from low-quality feedstocks to improve thermochemical conversion processes, prevent equipment corrosion and fouling caused by ash build-up, as well as to reduce biomass buffering capacity and improve pretreatment efficiency [6–8, 16]. Preprocessing, however, has also been proposed as a method to remove other types of NSC, such as acetic acid and extractives. Previous work from our research group found that dilute-acid preprocessing of poplar WTC resulted in higher monomeric sugar yield in the liquid fraction after pretreatment, improved cellulose digestibility during enzymatic hydrolysis, and increased ethanol fermentation yield from 5 to 55% due to the removal of NSCs during preprocessing [17]. Similarly, Castro et al. investigated the effects of alkaline deacetylation on xylose fermentation using *Scheffersomyces stipitis* and reported that the removal of acetyl groups from the biomass led to an increase in the fermentation yield from 0.25 to 0.37 g/g without any detoxification [18]. The major drawback of preprocessing, however, is the large water usage during the washing, which has a major impact on the overall operating cost. Economic analyses reported by previous studies suggested the use of recycled process water for the washing step to make it more cost-effective [17, 19].

This work aims to solve the discussed problems above by developing a novel lignocellulosic ethanol production process that increases the ethanol yield and simultaneously reduces the production cost. First, a technical assessment will be performed with the intent to (1) compare two types of poplar feedstock (WTC and CPC), in which CPC will be considered as a base case scenario due to its superior quality; (2) test three different preprocessing conditions (acidic, alkaline, and neutral) and assess their impacts on final ethanol yield; (3) assess if preprocessing can replace the detoxification step without compromising ethanol yields. Second, an economic assessment will be performed focusing on three large-scale biorefinery process scenarios, where the key points of comparison are the type/price of feedstock (WTC versus CPC) and the process configuration (preprocessing versus overliming). The main goal of this economic assessment is to determine which scenario promotes greater revenue along with lower costs. To achieve this, our study will (1) determine the annual revenue associated with the ethanol production capacity of each scenario; (2) compare the costs associated with the type of feedstock and process configuration; (3) assess the economic impact of using process water in the preprocessing step.

## Results and discussion

First, this study performed a technical assessment of preprocessing to investigate its impacts on the overall bioconversion yields using both feedstocks poplar whole-tree chips (WTC) and clean pulp chips (CPC). Total monomeric sugar yield (kilograms of monomeric sugars obtained per tonne of OD raw biomass) and recovery (percentage of monomeric sugars recovered from original sugars) after steam pretreatment and enzymatic hydrolysis were determined, and the ethanol fermentation yields of untreated and preprocessed samples were compared. It was also assessed if preprocessing could replace overliming detoxification in the ethanol bioconversion process. All percentage increase/decrease presented in this discussion were calculated based on the untreated biomass as the original value. Any data analysis mentioned as “significant” represents statistically significant (p < 0.05). Second, an economic assessment was performed on three large-scale biorefinery scenarios: (1) CPC feedstock via pretreatment, enzymatic hydrolysis, overliming, and sugars to ethanol fermentation (base case scenario), (2) WTC feedstock via preprocessing, pretreatment, enzymatic hydrolysis, and sugars to ethanol fermentation, and (3) CPC feedstock via preprocessing, pretreatment, enzymatic hydrolysis, and sugars to ethanol fermentation.

### Technical assessment

#### Chemical composition of untreated and preprocessed biomass

Table 1 shows the chemical composition of untreated (original) and preprocessed poplar whole-tree chips (WTC) and clean pulp chips (CPC).

**Table 1:** Chemical composition of untreated and preprocessed poplar biomass (as a percentage of the OD weight)

	Ash (%)	Extractives (%)	Glucan (%)	Xylan (%)	Total sugars* (%)	Total lignin (%)	Acetic acid (%)
Whole-tree chips (WTC)							
Untreated	1.6 ± 0.1^a^	10.7 ± 0.1^a^	42.1 ± 0.9^a^	14.3 ± 0.3^a^	60.6 ± 1.2^ab^	28.6 ± 0.8^a^	4.8 ± 0.2^a^
Acidic	0.5 ± 0.1^b^	4.6 ± 0.3^b^	41.4 ± 0.9^a^	15.3 ± 0.2^b^	59.5 ± 1.2^bc^	28.1 ± 0.2^ab^	5.7 ± 0.7^a^
Alkaline	1.6 ± 0.1^a^	4.3 ± 0.4^b^	42.5 ± 0.5^a^	15.6 ± 0.1^b^	61.8 ± 0.7^a^	27.6 ± 0.2^ab^	2.7 ± 0.1^b^
Neutral	1.4 ± 0.1^c^	6.8 ± 0.2^c^	39.6 ± 0.3^b^	14.6 ± 0.1^a^	57.9 ± 0.5^c^	27.4 ± 0.2^b^	5.2 ± 0.1^a^
Clean pulp chips (CPC)							
Untreated	0.6 ± 0.0^a^	3.5 ± 0.3^a^	47.9 ± 0.4^a^	14.4 ± 0.1^a^	64.7 ± 0.7^a^	26.1 ± 0.1^a^	5.0 ± 0.6^a^
Acidic	0.1 ± 0.1^b^	2.0 ± 0.1^b^	48.4 ± 1.3^a^	14.4 ± 0.4^a^	64.9 ± 1.7^a^	26.1 ± 0.2^a^	4.4 ± 0.2^a^
Alkaline	0.8 ± 0.1^c^	3.0 ± 0.0^c^	52.4 ± 0.9^b^	15.7 ± 0.3^b^	70.7 ± 1.3^b^	25.8 ± 0.1^a^	1.4 ± 0.1^b^
Neutral	0.4 ± 0.0^d^	2.6 ± 0.1^d^	49.4 ± 0.7^a^	14.9 ± 0.3^a^	66.8 ± 1.0^a^	26.3 ± 0.5^a^	4.8 ± 0.1^a^

Data represented as the mean values of triplicate analysis with standard deviation, extractives as duplicates

Different superscript letters indicate statistically significant differences (*p* < 0.05) within each column by Tukey’s test (WTC and CPC treatments were compared separately)

*Total sugars include glucan, xylan, arabinan, galactan, and mannan

#### Non-structural components (ash and extractives)

Untreated WTC showed greater non-structural components (NSC) content (12% ash plus extractives) than CPC (4%) (Table 1) due to the chemical composition of different parts of the tree. The white wood fraction of untreated WTC contained approximately 7% NSC (including ash and extractives), while the bark fraction contained 35% (Supplementary Material, Table S.1). This finding agrees with earlier reports by Passialis et al., where they stated that bark of black locust has higher ash and extractives content than other wood components [20]. Dou et al. [3] also compared the chemical characteristics of different fractions of 2-year-old poplar, and they reported that extractives content in bark was about two times higher than that in white wood, while ash content in bark was about five times higher than that in white wood. Furthermore, previous studies reported that juvenile wood has higher extractives content than mature wood [21]. Since WTC poplar was harvested at a younger age than CPC, it can be inferred that WTC has a higher juvenile wood content than CPC [22]. In good agreement with those studies, the white wood fraction of untreated WTC had 6.3% extractives (Supplementary Material, Table S.1), while the CPC had 3.5%.

Among the preprocessing conditions, acidic preprocessing was the most effective in removing ash from both WTC and CPC biomass (66% and 81% removal, respectively), followed by neutral preprocessing (11% and 32% removal, respectively) (Table 1). These findings are consistent with the literature, where Hӧrhammer et al. [17] reported 59% ash removal from poplar WTC using an acidic-neutral wash, and He et al. [6] reported 20% ash removal after neutral washing of corn stover. Interestingly, alkaline preprocessed CPC biomass showed an approximate 40% increase in total ash content when compared to untreated CPC. This finding can be explained by an accumulation of sodium cations originated from the sodium hydroxide solution used during alkaline preprocessing (see results in Table 2). Although Kundu et al. [23] and Cho et al. [24] also studied the deacetylation of homogeneous yellow poplar biomass using dilute sodium hydroxide solution, they did not report its effects on the NSC content. Differently from CPC, the ash content of WTC did not significantly change (*p* < 0.05) after alkaline preprocessing, showing only a 3% increase when compared to untreated.

**Table 2:** Elemental composition of untreated and preprocessed poplar biomass

	Ca (µg/g)	K (µg/g)	Mg (µg/g)	Na (µg/g)	P (µg/g)	S (µg/g)
Whole-tree chips (WTC)						
Untreated	3140 ± 142	1893 ± 25	429 ± 10	0.0 ± 0.0	560 ± 35	228 ± 10
Acidic	1776 ± 56	0.0 ± 0.0	0.0 ± 0.0	0.0 ± 0.0	183 ± 8	152 ± 5
Alkaline	3248 ± 90	211 ± 19	358 ± 4	692 ± 0.0	250 ± 25	123 ± 7
Neutral	3256 ± 77	1015 ± 27	391 ± 23	0.0 ± 0.0	495 ± 11	196 ± 4
Clean pulp chips (CPC)						
Untreated	831 ± 28	793 ± 12	237 ± 19	0.0 ± 0.0	170 ± 5	77 ± 2
Acidic	140 ± 6	0.0 ± 0.0	0.0 ± 0.0	0.0 ± 0.0	0.0 ± 0.0	66 ± 2
Alkaline	778 ± 52	0.0 ± 0.0	225 ± 20	1594 ± 24	57 ± 3	50 ± 6
Neutral	744 ± 20	0.0 ± 0.0	157 ± 10	0.0 ± 0.0	108 ± 14	70 ± 4

Data represented as the mean values of duplicate analysis with standard deviation

Other elements were analyzed (including barium, iron, manganese, and silica), but they were either not detected or they were present at trace amounts (lower than 50 µg/g)

Similarly, acidic preprocessing removed extractives to a greater extent, with 57% and 42% extractives removal from WTC and CPC biomass, respectively (Table 1). Neutral preprocessing removed 37% and 25% extractives from WTC and CPC, respectively. Similar results were reported by Hӧrhammer et al. [17], where acidic-neutral and neutral washes removed 43% and 51% extractives from poplar WTC, respectively.

#### Total sugars, lignin, and acetic acid

The total sugars of both types of biomass presented minor changes when comparing untreated and preprocessed samples (Table 1), with ranges of 57.9%–61.8% total sugars for WTC and 64.8%–70.7% for CPC. The fact that preprocessing did not compromise the sugar content to a big extent is favorable for its application in ethanol production. Similarly, the total lignin content also showed minimal changes after preprocessing, with numbers ranging from 27.4 to 28.6% total lignin content for WTC and 25.8%–26.3% for CPC. Acetic acid, however, was extensively removed by alkaline preprocessing (48% and 73% from WTC and CPC, respectively). This finding is consistent with those from Chen et al. [19], where deacetylation removed 80% of acetyl groups from corn stover. No significant (*p* < 0.05) removal of acetic acid was obtained with acidic and neutral conditions.

#### Elemental composition

Elemental analysis was performed to characterize the mineral composition of untreated and preprocessed biomass (Table 2). Calcium and potassium were predominant in both untreated WTC (3140 µg/g and 1893 µg/g, respectively) and CPC (831 µg/g and 793 µg/g, respectively), followed by magnesium, phosphorus, and sulfur. These findings are consistent with the literature, where the main inorganic components found in woody biomass are calcium, potassium, and magnesium [22]. As expected, acidic preprocessing was more effective in removing minerals from both biomass: potassium and magnesium were completely removed, while calcium was partially removed (43% and 83% removal from WTC and CPC, respectively). Calcium is present in the biomass in different forms, such as acid-soluble salts, non-leachable salts, and organically bound metal ions which are very difficult to be removed [25]. Alkaline preprocessing did not remove calcium but removed 89% and 100% of potassium from WTC and CPC, respectively. Not surprisingly, alkaline preprocessing added sodium to both WTC and CPC (692 µg/g and 1594 µg/g, respectively) due to sodium hydroxide diffusion into the wood during preprocessing, and the sodium cations bound to acid groups in the wood matrix.

#### Buffering capacity

**Fig. 1:**
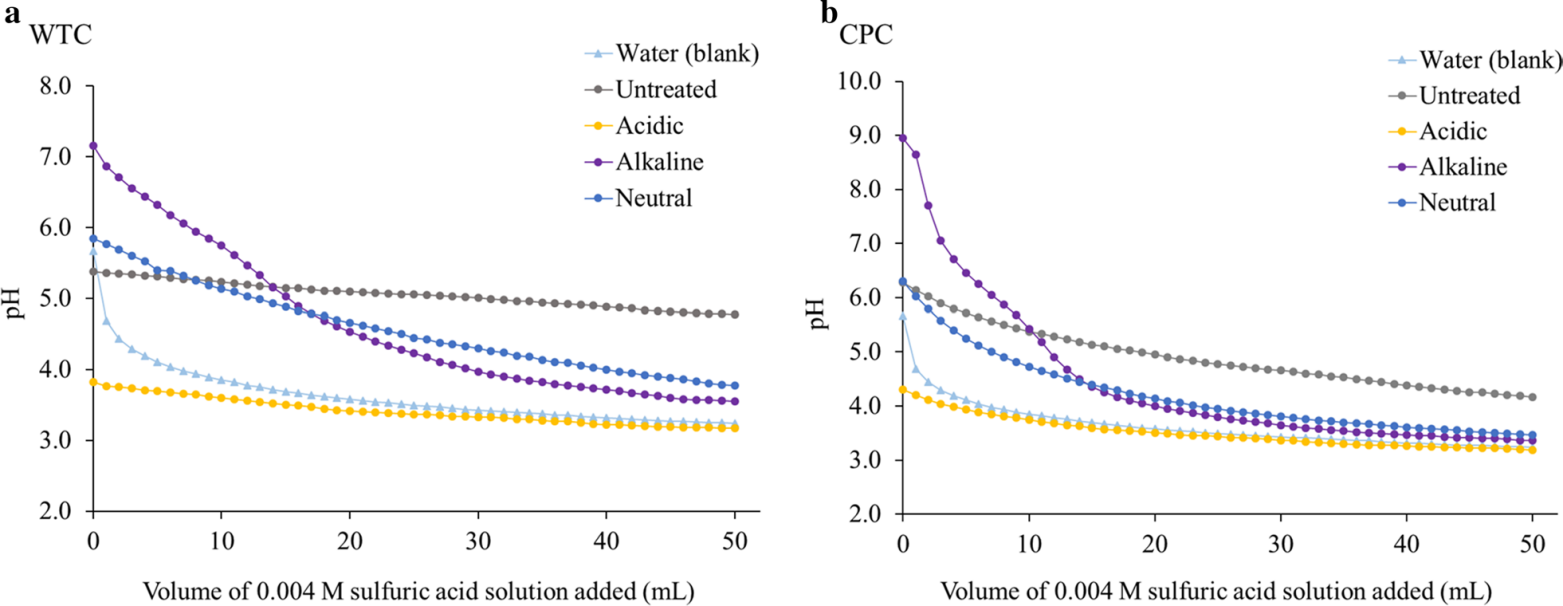
Titration curves of untreated and preprocessed poplar biomass of both **a** WTC samples and **b** CPC samples, along with DI water (blank) as a reference

Buffering capacity of untreated and preprocessed biomass was measured to determine how the biomass pH changes with the addition of a dilute acid solution. The steam pretreatment is usually carried out under acidic conditions and it has been suggested that ash can buffer the pH reduction during pretreatment, consequently decreasing the pretreatment efficacy [26]. Figure 1 shows the titration curves for water extracts of untreated and preprocessed biomass, and DI water was used as a reference. Different preprocessing conditions had different initial pH for both WTC and CPC biomass due to the presence of residual chemicals from the preprocessing step. The pH of untreated WTC extract stayed quite stable with the continuous addition of a dilute-acid solution (pH dropped from 5.4 to 4.8), reflecting the high buffering capacity of the untreated biomass due to its higher ash content (Fig. 1a). Acidic preprocessed WTC biomass displayed a similar behavior as the water blank, indicating a lower buffering capacity as a result of its low total ash content (Table 1). Alkaline preprocessed WTC started at pH 7.2 due to the presence of residual caustic from preprocessing, and the pH drop during the addition of the first 30 mL of acid solution illustrates the occurrence of neutralization reactions. Once the pH of all samples was stable, it was noted that all preprocessing conditions were able to reduce the buffering capacity of the biomass and achieve a lower pH than untreated biomass. Hörhammer et al. had similar results, where acidic and neutral preprocessing decreased the buffering capacity of 2-year-old poplar whole-tree chips when compared to untreated biomass due to a lower ash content [17]. The CPC samples presented similar trends as the WTC, as it is shown in Fig. 1b. Alkaline preprocessed CPC started at a higher pH 9 as a result of greater residual caustic content in the biomass (1594 µg/g) when compared to the alkaline WTC sample (692 µg/g).

#### Liquid and solid fractions after steam pretreatment

##### Chemical composition of liquid fraction

Most sugars recovered in the liquid fraction after pretreatment were xylose, representing 50–70% of the total sugars (varying with the preprocessing condition), followed by glucose, which corresponded to 19–40%. Minor sugars, such as arabinose, galactose, and mannose were present at trace amounts. Table 3 shows the total sugar yield (kg/tonne of biomass) in the liquid fraction and the corresponding percentage of sugar present in monomeric form. For poplar WTC, acidic and neutral preprocessing had the highest total sugar yield, 258 and 257 kg/tonne, respectively, thus obtaining approximately 8% more kilograms of sugars per tonne of biomass than untreated WTC. 91% of the total sugar released in the acidic preprocessed WTC liquid fraction was in monomeric form, approximately 12% greater than all other samples, including untreated biomass. Previous studies reported that the removal of NSC from biomass can enhance the hydrolysability of hemicellulose during the pretreatment step as a result of a lower buffering capacity effect [6, 17]. In good agreement with those studies, acidic preprocessing showed the highest removal of NSC (66% removal of ash) and lower buffering capacity, which improved the hemicellulose hydrolysis into monomers.

**Table 3:** Chemical composition of liquid and solid fractions after steam pretreatment, and enzymatic hydrolysis conversion

	Liquid fraction			Solid fraction						
				Chemical composition				Elemental composition^3^		Enzymatic hydrolysis
	pH	Total sugars^1^		Glucan	Xylan	Lignin	Ash	Ca	S	Glucose conversion^4^
		kg/tonne	mono %^2^	%	%	%	%	µg/g	µg/g	%
Whole-tree chips (WTC)										
Untreated	1.8	239 ± 7	79.0%	60.7 ± 0.5	2.4 ± 0.0	36.6 ± 0.3	1.0 ± 0.3	3000 ± 171	1062 ± 13	67.7 ± 1.4
Acidic	1.4	258 ± 2	91.1%	62.7 ± 0.1	1.3 ± 0.0	38.4 ± 0.9	0.2 ± 0.0	1349 ± 55	811 ± 22	75.7 ± 1.2
Alkaline	1.6	245 ± 5	78.7%	63.1 ± 0.7	2.6 ± 0.0	35.2 ± 0.1	0.5 ± 0.2	2315 ± 10	1084 ± 16	72.7 ± 1.2
Neutral	1.6	257 ± 9	78.9%	61.9 ± 0.5	1.6 ± 0.1	37.6 ± 0.3	1.0 ± 0.1	2766 ± 81	1144 ± 19	72.8 ± 0.6
Clean pulp chips (CPC)										
Untreated	1.4	168 ± 5	96.2%	68.0 ± 1.1	1.2 ± 0.1	34.4 ± 0.4	<0.1%	126 ± 16	486 ± 20	78.2 ± 2.3
Acidic	1.4	213 ± 4	101.2%	66.8 ± 1.1	0.0 ± 0.0	33.6 ± 0.2	<0.1%	97 ± 19	408 ± 12	76.7 ± 1.2
Alkaline	1.6	176 ± 1	93.4%	72.9 ± 0.6	2.4 ± 0.0	28.3 ± 0.2	<0.1%	99 ± 10	562 ± 21	69.8 ± 1.4
Neutral	1.5	201 ± 5	93.5%	70.6 ± 0.3	1.4 ± 0.1	31.5 ± 0.5	<0.1%	57 ± 6	431 ± 11	73.2 ± 0.1

Data represented as the mean values of triplicate analysis (except elemental composition that was done in duplicate), which in turn comes from two steam pretreatments replicates

^1^ Total sugars (kg/tonne of original biomass) include glucan, xylan, arabinan, galactan, and mannan

^2^ Monomeric sugar percentage of the total sugars

^3^ Magnesium, potassium, phosphorus, and sodium were completely removed (0 µg/g) during steam pretreatment from both WTC and CPC. Other elements were analyzed (including barium, iron, manganese, and silica), but they were either not detected or they were present at trace amounts (lower than 50 µg/g)

^4^ Maximum glucose conversion: WTC samples after 96 h of hydrolysis, and CPC samples after 48 h

For CPC, acidic preprocessing had the highest total sugar yield of 213 kg/tonne of biomass, approximately 27% higher than untreated biomass (168 kg/tonne). Unlike WTC, all CPC samples had a monomeric sugar percentage above 93% as a result of the original low NSC of this biomass, with the highest percentage of ~ 100% for acidic preprocessing. Interestingly, CPC liquid fractions showed lower total sugar yield than WTC liquid fractions, which being possibly related to differences in anatomical properties between the WTC and CPC wood fibers. According to Bao et al., fibers in juvenile wood are about 24% shorter than those in mature wood, therefore, being more susceptible to fractionation during steam pretreatment and resulting in better hemicellulose solubilization [21].

##### Chemical composition of solid fraction and enzymatic hydrolysis conversion

Table 3 also shows the chemical composition of the solid fractions and maximum cellulose to glucose conversion after enzymatic hydrolysis (EH) of WTC (after 96 h of reaction) and CPC solids (after 48 h of reaction). For WTC, all preprocessed samples had higher EH conversions than untreated. Untreated WTC had the lowest conversion of 68% as a result of the combination of higher xylan and ash contents (2.4% and 1.0%, respectively) when compared to the preprocessed samples. Previous studies [27–29] have reported that xylan has a negative effect on cellulose digestion because it behaves like a physical barrier blocking the access of enzymes to the cellulose fibers. Furthermore, He et al. [6] and Bin [30] reported that certain cations, including calcium, can negatively affect the hydrolysis by inhibiting the activity of endoglucanases and exoglucanases. Accordingly, acidic preprocessed WTC resulted in the highest conversion of 76% (8% improvement when compared to untreated WTC) due to its lower xylan and ash contents (1.3% and 0.2%, respectively). The lower xylan content in acidic preprocessed solids is associated with greater solubilization of the hemicellulose during pretreatment [31].

In general, the EH of all CPC samples was faster than that of WTC, which may be associated with the overall lower ash and lignin content of CPC solids. Surprisingly, alkaline preprocessed CPC solids had the lowest EH conversion (70% after 48 h of hydrolysis) among all CPC samples (Table 3). The yields (kg/tonne) of sugars, lignin, and ash of the solid fractions are shown in Supplementary Material, Table S.3.

The elemental composition of the solid fraction is also presented in Table 3. Potassium, magnesium, sodium, and phosphorus were utterly removed in all WTC and CPC samples during steam pretreatment. Calcium, conversely, was removed to a lesser extent due to its lower solubility [25]. Overall, WTC solids showed lower calcium removal during pretreatment than CPC solids. Compared to the original calcium content of each sample before pretreatment (Table 2), alkaline preprocessed WTC had 29% removal, followed by acidic with 24% removal, neutral with 15%, and finally untreated WTC with a minor removal of 4%. In contrast, untreated CPC solids showed a calcium removal of 85%, which is about 20 times greater than the removal in untreated WTC solids. Neutral preprocessing had the highest calcium removal of 92%. The difference in calcium removal between WTC and CPC biomass can be attributed to the presence of bark in WTC, which is where the majority of calcium is encountered (Supplementary Material, Table S.2). Finally, an increase in sulfur content when compared to samples before pretreatment (Table 2) was observed in the order of 4 to ninefold for WTC and 6 to 11-fold for CPC, which was originated from the SO_2_ used during biomass impregnation.

#### Monomeric sugar recovery and yield after preprocessing, steam pretreatment, and enzymatic hydrolysis

**Fig. 2:**
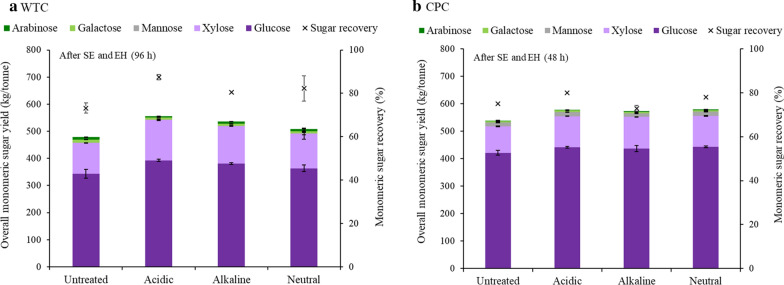
Total monomeric sugar recovery and yield of **a** WTC samples and **b** CPC samples

Figure 2 illustrates the total monomeric sugar recovery (percentage of monomeric sugars recovered from original sugars) and yield (kilograms of monomeric sugars obtained per tonne of OD raw biomass) after steam explosion (SE) and enzymatic hydrolysis (EH) for both WTC (Fig. 2a) and CPC (Fig. 2b). Error bars indicate standard deviation from duplicate measurements. The complete data, including statistical analysis, can be found in the Supplementary Material, Table S.3.

The monomeric sugar recovery of WTC significantly (*p* < 0.05) improved when preprocessing was done (Fig. 2a). Acidic preprocessing achieved remarkable 87.5% monomeric sugar recovery, while untreated WTC had a recovery of 73%. Neutral and alkaline preprocessed WTC had monomeric sugar recovery of 82% and 81%, respectively. CPC samples presented only minor differences in monomeric sugar recovery (Fig. 2b), with the highest being achieved by acidic preprocessing (80%), only 5% higher than untreated CPC. Overall, WTC samples recovered more monomeric sugars after SE and EH than CPC samples, which could be related to the different morphology of the biomass and its effects on hemicellulose solubilization during pretreatment, as discussed previously.

In like manner, Fig. 2a shows that untreated WTC had the lowest monomeric sugar yield of 493 kg/tonne, while WTC preprocessed under acidic condition resulted in a significantly (*p* < 0.05) higher yield of 578 kg/tonne, followed by alkaline (553 kg/tonne) and neutral (529 kg/tonne). Hӧrhammer et al. [17] reported similar results, where the acidic wash of poplar WTC resulted in a 90 kg increase in monomeric sugar yield when compared to untreated. For the CPC samples (Fig. 2b), acidic (577 kg/tonne), alkaline (573 kg/tonne), and neutral (580 kg/tonne) preprocessing demonstrated significant (*p* < 0.05) improvement in monomeric sugar yield when compared to untreated (539 kg/tonne). There was no statistical difference (*p* < 0.05) in monomeric sugar yield between the three CPC preprocessing conditions.

#### Fermentation and detoxification

Ethanol fermentation was performed separately on the liquid fraction after pretreatment and the liquids resulted from EH of solids. The initial concentrations of sugars (glucose and xylose) and maximum concentration of ethanol obtained in the fermentation experiments can be found in the Supplementary Material, Table S.5. An additional detoxification step was performed on the CPC liquid fractions after pretreatment prior to fermentation to simulate the base case scenario of an ethanol biorefinery. Ammonia conditioning, as described in the NREL 2011 report [15], was first tested as the detoxification step. However, this method was not effective in poplar CPC liquid fractions, resulting in an unfermentable liquid (ethanol fermentation yield of only 1%—data not shown). It appears that ammonia conditioning might not be able to remove certain types of fermentation inhibitors present in poplar liquid fraction after pretreatment. This inference goes along with a research study by Persson et al. [32], which reported that conditioning spruce liquid fraction after pretreatment to pH 10 was far more effective in removing inhibitors (mostly furfural, HMF, and phenols) than only neutralizing to pH 5.5 using four different bases (NaOH, KOH, Ca(OH)_2_, and NH_3_). For this reason, the present study chose the well-established overliming as the one-step detoxification method used for the CPC liquid fractions after pretreatment, based on the NREL 2002 report practices [10].

##### Fermentation of solids after enzymatic hydrolysis

Since the liquid obtained after EH of the solid fraction contained only monomeric sugars and no inhibitors, all WTC and CPC samples reached a maximum ethanol conversion after 8 h of reaction with very similar yields ranging from 82 to 85% (Table 4).

**Table 4:** Ethanol fermentation yields of solid fraction after EH and liquid fraction after pretreatment (with and without overliming), and total sugar loss after with overliming

		Ethanol fermentation yield^1^ (Y _%T_)			
		Solid fraction after EH	Liquid fraction	Overlimed liquid fraction	Total sugar loss after overliming^3^ (%)
	Control^2^	84.6 ± 3.8	79.1 ± 1.6	77.7 ± 2.1	NA
Whole-tree chips (WTC)	Untreated	82.2 ± 2.7	1.9 ± 0.2	NA	NA
	Acidic	82.8 ± 0.6	53.2 ± 1.3	NA	NA
	Alkaline	82.3 ± 0.9	55.0 ± 0.2	NA	NA
	Neutral	83.4 ± 0.6	49.0 ± 0.4	NA	NA
Clean pulp chips (CPC)	Untreated	83.7 ± 0.8	1.1 ± 0.0	45.3 ± 0.5	29.5
	Acidic	82.5 ± 1.4	52.0 ± 3.2	53.3 ± 1.0	19.4
	Alkaline	81.8 ± 1.7	55.1 ± 1.5	64.6 ± 0.6	20.4
	Neutral	85.5 ± 2.4	55.6 ± 1.9	54.6 ± 1.3	19.2

Data represented as the mean values of duplicate analysis with standard deviation

NA = Not Applicable

^1^ Ethanol fermentation yield is expressed as a percent of theoretical yield (Y _%T_), according to Eq. 1

^2^ The fermentation control contained reagent-grade sugars at similar concentrations to those in experimental samples

^3^ Accounting for glucose and xylose only (main sugars present in the liquid fraction after steam pretreatment)

##### Fermentation of liquid fractions

General trends were observed in fermentation yields of both WTC and CPC liquid fractions after pretreatment without detoxification (Table 4). First, untreated samples resulted in negligible ethanol yields (1.9% and 1.1% of theoretical ethanol yield for untreated WTC and CPC, respectively). In contrast, liquid fractions from preprocessed WTC and CPC presented a significant improvement in ethanol conversion, with yields ranging from 49 to 56%. This finding could be associated with the removal of specific types of extractives with antimicrobial characteristics during preprocessing (Table 1), which were inhibiting the fermentation of untreated samples [9, 33]. Similar trends were reported by Hӧrhammer et al. [17], who observed a 50% increase in ethanol yield when acidic preprocessing was performed with 2-year-old whole-tree chips poplar biomass.

##### Fermentation of overlimed CPC liquid fractions

Since CPC was chosen as the base case feedstock due to its superior quality, overliming was performed in these liquid fraction samples to replicate existing ethanol biorefinery models. Not surprisingly, overliming increased the ethanol fermentation yield of untreated CPC from 1.1 to 45% (Table 4). The fermentation yields of acidic and neutral preprocessed CPC samples with overliming did not demonstrate improvements compared to the samples without overliming (53% and 55% ethanol yield, respectively). In contrast, alkaline preprocessed CPC sample had a 10% improvement on fermentation yield when overliming was done. It should be highlighted that, when comparing the increase in CPC fermentation yield exclusively via overliming (from 1.1% to 45%) with that exclusively via preprocessing (from 1.1 to 52–55%), similar improvements were observed by both methods. Most importantly, overliming resulted in a sugar loss of approximately 30% in untreated CPC and 19–20% in all preprocessed CPC samples when compared to the original sugar content in the liquid fractions (Table 4). Hence, even though the fermentation yield increased with overliming (in the case of untreated and alkaline preprocessed CPC), the associated sugar loss results in a reduction of the final ethanol production per tonne of biomass. Consequently, preprocessing alone seems to be a better option than overliming because it improves the ethanol fermentation yield to similar extents without compromising the initial concentration of sugars.

Finally, it should be noted that the present study used non-genetically engineered microorganisms for the fermentation experiments, thus the fermentation yields shown in Table 4 were not optimized. NREL, for example, commonly uses recombinant co-fermenting bacteria to maximize the yield [15]. Henceforth, in an actual large scale biorefinery, where the fermentation is optimized, the concentration of monomeric sugars available for fermentation is the determining factor for the final ethanol production yield. With this in mind, this study demonstrated that acidic preprocessing could substantially improve the ethanol production of a biorefinery because of its higher monomeric sugar yield using both types of poplar biomass.

### Economic assessment

#### Large-scale biorefinery ethanol production

Among all scenarios investigated so far, three of them were chosen to be further assessed regarding ethanol production in a large-scale biorefinery. The chosen scenarios were: (1) CPC feedstock via pretreatment, enzymatic hydrolysis, overliming, and ethanol fermentation (as a base case scenario); (2) WTC feedstock via acidic preprocessing, pretreatment, enzymatic hydrolysis, and ethanol fermentation (to assess the effects of preprocessing using biomass with high NSC content); and (3) CPC feedstock via acidic preprocessing, pretreatment, enzymatic hydrolysis, and ethanol fermentation (to assess the effects of preprocessing using biomass with low NSC content). This way, it is possible to determine the effects of preprocessing using both types of biomass and compare it to the base case using overliming detoxification.

**Fig. 3:**
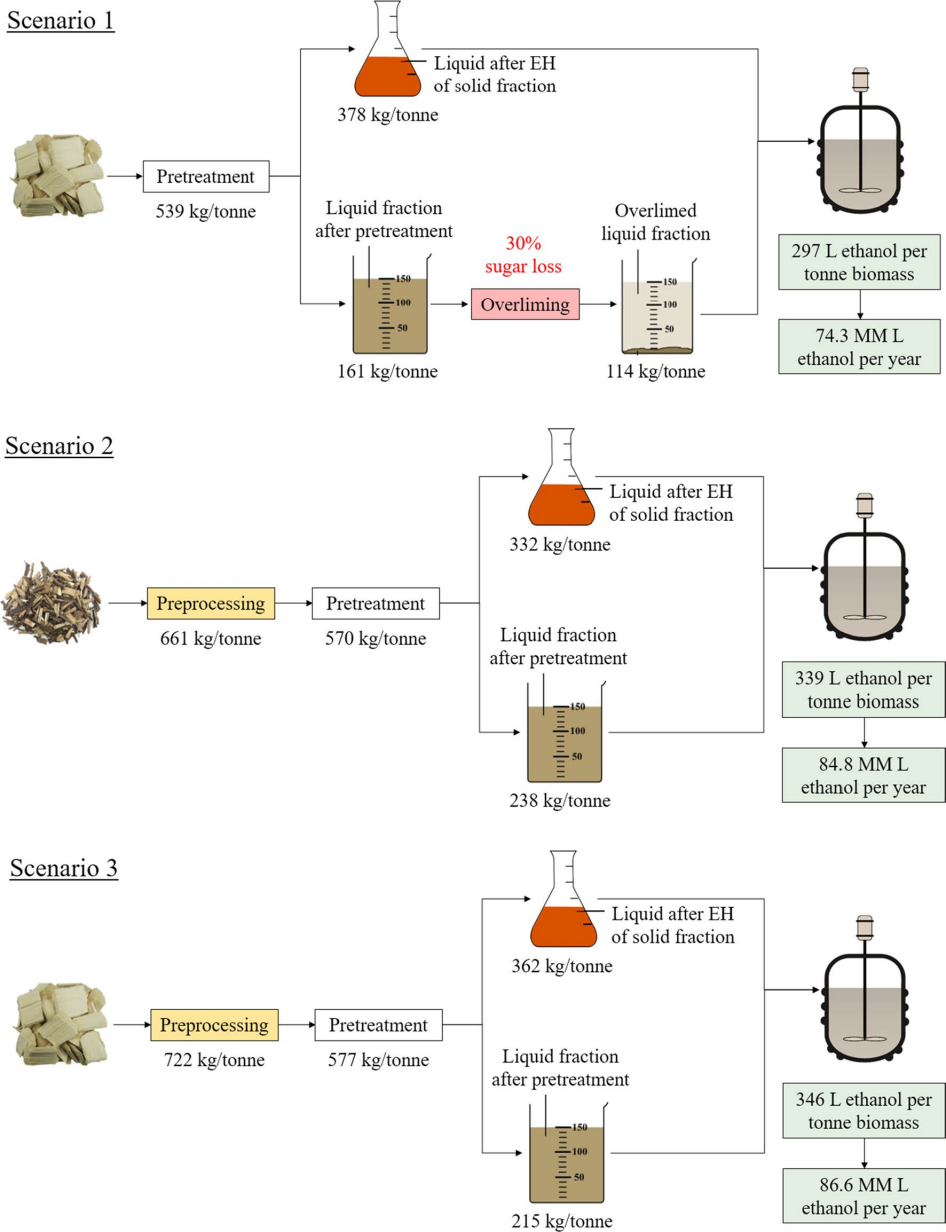
Annual large-scale ethanol production of the three scenarios assessed

Figure 3 shows the ethanol yield (liters per tonne of biomass) of each scenario calculated based on the total monomeric sugar yield obtained in the experimental part of this work (Fig. 2) from both liquid and solid fractions after SE and EH. For this assessment, the fermentation was assumed to be performed using a recombinant co-fermenting bacteria with 95% glucose conversion and 85% xylose conversion to ethanol [15]. Next, the large-scale biorefinery ethanol production (million liters per year) was calculated by combining the ethanol yield and a feedstock usage of 250,000 dry tonnes/year (Fig. 3).

It can be seen in Fig. 3 that the base case scenario 1 using poplar CPC as feedstock had the lowest ethanol production (74.3 MM L/year) mostly due to the 30% sugar loss in the liquid fraction after pretreatment associated with overliming. Scenario 2, on the other hand, used poplar WTC as feedstock with acidic preprocessing and no overliming, resulting in ethanol production of 84.8 MM L/year. This increase of 10.5 MM L/year compared to scenario 1 was due to both a higher amount of monomeric sugars available for fermentation and no sugar loss resulting from overliming. Similarly, scenario 3 used CPC via acidic preprocessing and resulted in the highest ethanol production of 86.6 MM L/year as a result of using low-NSC biomass with higher initial sugar content. It is clear that acidic preprocessing has the potential to increase the ethanol production of a large scale biorefinery using both types of poplar feedstocks. To have a more complete picture of the economics of each scenario, this study compared the costs of the unit process involved (either preprocessing or overliming) and the different feedstocks used.

#### Cost assessment of preprocessing and overliming

First, the capital costs were assessed: the overliming unit operation was assumed to be the same as the one in the 2002 NREL report [10], while the preprocessing unit was assumed to have a similar configuration as the deacetylation process from the 2015 NREL report [34]. Chemical Engineering Plant Cost Index (CEPCI) was used to properly adjust the price of the equipment to the year 2018, and a scaling exponent of 0.6 was used to adjust the capital cost of each equipment to our biorefinery size. The biorefinery size ratio was calculated based on the feedstock consumption rate (dry tonne/day). Second, the direct operating costs of both unit processes were calculated: the direct operating cost of overliming included chemicals (calcium hydroxide and sulfuric acid) and gypsum disposal to a landfill, while the direct operating cost of acidic preprocessing included water and sulfuric acid for a final liquid-to-biomass ratio of 4:1. All operating costs were calculated based on the latest pricing quotes and properly scaled to the biorefinery flow rates. The complete individual equipment and chemical prices used can be found in the Supplementary Material, Tables S.6 and S.7.

The calculated capital and direct operating costs of both processes are presented in Table 5. It can be seen that the capital cost of preprocessing was about twice higher than that for overliming, while the direct operating cost of preprocessing using fresh water was approximately 4 times higher than that of overliming due to the high cost of fresh water. Chen et al. [19] performed a techno-economic analysis of deacetylation of corn stover using a liquid-to-biomass ratio of 3:1, and for every gallon of ethanol produced their process required 3.5–4.5 gallon of fresh water, thus increasing the total costs. For this reason, the present study also considered the use of process water recycled from the system as a more cost-effective alternative for preprocessing, which lowered its direct operating cost by 45-fold (Table 5). The source of process water will be further discussed in the section “Proposed process model”.

**Table 5:** Capital and operating costs of overliming and preprocessing units

	Capital cost (MM $)	Direct operating cost with fresh water (MM $/year)	Direct operating cost with process water (MM $/year)
Overliming	$ 1.38	$ 0.96	$ 0.96
Preprocessing	$ 2.68	$ 4.18	$ 0.16

#### Overall cost assessment of scenarios

It is important to realize that Table 5 provides an incomplete picture of the overall process economics of the scenarios, since they use different feedstocks and have different ethanol production yields. With this in mind, first, the annual cost of each feedstock was calculated and added to the operating cost of each scenario (Table 6). Based on the feedstock usage of 250,000 dry tonnes/year, the annual feedstock cost of poplar CPC was calculated as $30.2 MM, while the cost of poplar WTC was $20.1 MM. Second, to define the annual revenue of each scenario, their final ethanol production presents in Fig. 3 (liters per year) was multiplied by the cellulosic ethanol selling price of $0.94 per liter. This price included the selling price of the fuel plus the cellulosic waiver credit (CWC) and D5 RIN, as regulated by the Renewable Fuel Standard program [35], and the CWC and D5 RIN prices were determined using the latest U.S. EPA 2019 guidelines [36]. A summary of the capital and total operating costs (including the feedstock cost and using process water for preprocessing), as well as the annual revenue of the three scenarios, are presented in Table 6. It can be noted that the total operating cost of scenario 2, using WTC feedstock via acidic preprocessing, was $11 million cheaper than the base case biorefinery scenario 1 because of the cheaper feedstock. Besides, preprocessing considerably increased the final annual revenue in scenarios 2 and 3 when compared to the base case scenario 1 by $9.8 and $11.5 million, respectively.

**Table 6:** Capital cost, total operating cost, and revenue of the three scenarios proposed

	Description	Capital cost (MM $)	Total operating cost* (MM $/year)	Annual revenue (MM $/year)
Scenario 1	CPC feedstock, with overliming	$ 1.38	$ 31.20	$ 70.02
Scenario 2	WTC feedstock, with acidic preprocessing using process water	$ 2.68	$ 20.24	$ 79.85
Scenario 3	CPC feedstock, with acidic preprocessing using process water	$ 2.68	$ 30.41	$81.53

* Total operating cost including the feedstock cost

Finally, to assess the economic benefits of switching from the base case scenario that uses CPC via overliming to the new proposed processes, the incremental return on investment (ROI) associated with making these process changes was calculated pairwise between scenarios 1 and 2, and between scenarios 1 and 3 (Eq. 2). The ROI between scenarios 1 and 2 was determined to be 1600%, meaning that there is an enormous return on using WTC feedstock via acidic preprocessing using process water instead of the base case process. This astounding ROI is due to four main reasons: (a) WTC feedstock is substantially cheaper than CPC, and the feedstock was the biggest contributor to the operating cost; (b) using process water in the preprocessing step significantly decreased the operating cost of preprocessing; (c) acidic preprocessing resulted in higher ethanol production and consequently higher revenue; (d) the absence of overliming prevented the sugar loss and its resulting lower ethanol yields. In like manner, the ROI between scenarios 1 and 3 was 948%, demonstrating that by keeping the same feedstock and just switching the process from overliming to preprocessing still results in much higher revenue.

#### Proposed process model

As has been noted, preprocessing is a superior approach to conditioning the liquid fraction after pretreatment for subsequent fermentation compared to overliming because it eliminates the large sugar loss, results in higher ethanol production, and enables the use of low quality, but much cheaper, biomass feedstock. It also should be noted that biomass preprocessing will frequently be necessary for a biorefinery to remove the dirt and grift from the feedstock that would erode the downstream process equipment. In the present work, we propose that the preprocessing be engineered such that it cleans the feedstock and eliminates the need for the overliming process.

**Fig. 4:**
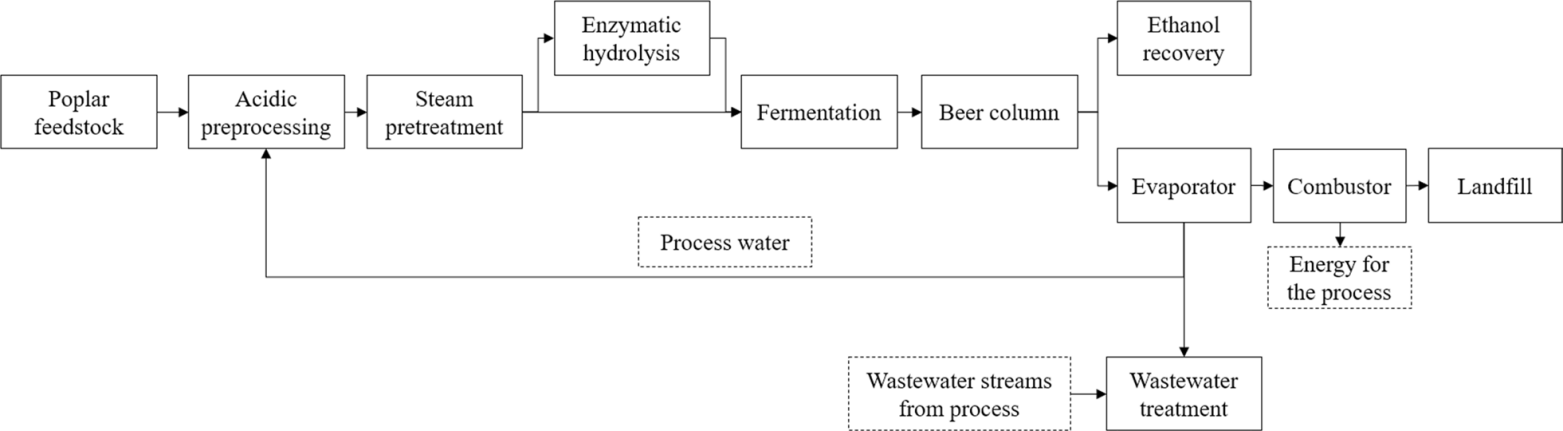
Simplified process flow of proposed biorefinery using poplar feedstock via acidic preprocessing using process water

A new process design was proposed by the authors based on the NREL 2002 design [10] with some key modifications (Fig. 4): a preprocessing unit was included prior to pretreatment, the overliming unit was removed, and process water was recycled to feed the preprocessing unit. Because of the simplicity of this process and the absence of ammonia conditioning, the evaporator design from the NREL 2002 report was maintained. In the proposed model, the evaporator had two main outlet streams: the stillage containing the organic and inorganic compounds in syrup form (which was directed to the combustor to generate electricity for the whole system), and the vapor condensate. The evaporator condensate stream was then directed to the preprocessing unit and contained enough water to reach the required liquid-to-biomass ratio of 4:1, while the excess of water was sent to the WWT plant. Because the evaporator was kept and the loading sent to WWT was minimized, the WWT plant in our process was assumed to be the same as the NREL 2002 design. According to NREL, this WWT configuration had an installed equipment cost of only $3.3 million [10]. Therefore, our research group believes that by substituting overliming by preprocessing not only solves the problem with sugar loss and gypsum formation but also enables the adoption of a much simpler and cheaper WWT design.

## Conclusions

The goal of this work was to develop an ethanol production process that could work with feedstocks with high and low NSC, but still, deliver good ethanol yield and thereby reduce production costs. The technical assessment of this study found that acidic preprocessing resulted in the highest monomeric sugar recovery after pretreatment and enzymatic hydrolysis of both WTC and CPC feedstocks. Acidic preprocessed WTC achieved 87.5% monomeric sugar recovery (14% higher than untreated WTC), while acidic preprocessed CPC had 80% recovery (5% higher than untreated CPC). This finding is related to ash removal, which led to a decrease in the biomass buffering capacity that resulted in better solubilization of sugars during pretreatment and in the improvement of the enzymatic hydrolysis conversion. Preprocessing also led to a significant increase in the liquid fraction fermentation yield from 1-2% (untreated) to 49-56%, similar to that achieved by overliming (45%). Hence, it was proven that preprocessing can substitute overliming and prevent unwanted sugar loss.

The economic assessment revealed that using poplar WTC as feedstock via acidic preprocessing and no overliming produced 10.5 million liters more ethanol per year than the base case scenario using CPC with overliming because of the higher monomeric sugar recovery and no sugar loss associated with overliming. Using process water instead of fresh water to feed the preprocessing unit reduced its operating cost by 45-fold. By switching from the base case scenario to the proposed process using WTC, an incremental return on investment of 1600% was obtained as a result of using cheaper feedstock, improvements on annual ethanol production via acidic preprocessing, and the significant decrease in the cost of the preprocessing when using process water. All things considered, this study has demonstrated that biomass preprocessing is effective while using both types of poplar feedstocks and has the additional benefit of substituting overliming. Even more, it was possible to develop a novel biorefinery process that promoted an increase in the ethanol yield while reducing the overall production costs. Finally, preprocessing enabled the use of evaporators in the downstream design that resulted in a waste stream with minimized loading, making possible the use of a less-complex WWT plant design.

## Methods

Two poplar feedstocks were used, poplar clean pulp chips (CPC) and poplar whole-tree chips (WTC), which were preprocessed under acidic, alkaline, or neutral conditions at 80 °C for 3 h. Next, untreated and preprocessed samples were steam-exploded (SE) at 195 °C for 5 min with 3% (w/w) SO_2_ impregnation, and the resulting slurry was vacuum filtered. Enzymatic hydrolysis (EH) of the solid fraction was performed at 50 °C, pH 4.8 for 96 h. A complete mass balance was conducted to determine the monomeric sugar yield and recovery after SE and EH. Both the liquid fraction after pretreatment and the liquid obtained after EH of the solid fraction were fermented at 30 °C, pH 6. Overliming was assessed as detoxification of CPC liquid fractions after pretreatment. Finally, an economic assessment was performed on three large scale biorefinery process scenarios to evaluate their annual revenue associated with ethanol production, the costs related to the type of feedstock (CPC versus WTC) and unit process (overliming versus preprocessing), and the impact of using process water for preprocessing.

### Raw materials

The two poplar feedstocks used (clean pulp chips and whole-tree chips) are hybrids of *Populus trichocarpa* and *Populus deltoids* obtained from a 7.8-acre plantation near Jefferson, OR managed by GreenWood Resources (Portland, OR). Both feedstocks comprise of thousands of poplar trees that were harvested using a modified forage harvester, as described in a previous study [3], and chipped in fall 2016 under different harvesting systems. The clean pulp chips (CPC) comprised of 12-year-old poplar trees that were harvested, cleaned, and debarked, resulting in a homogeneous feedstock comprised exclusively of white wood chips. The poplar whole-tree chips (WTC) comprised of 3-year-old trees harvested without leaves and chipped, resulting in a heterogeneous mixture of white wood (stem), bark, and branches. Both raw materials were stored at − 20 °C until used.

### Preprocessing

Preprocessing was carried out using three different solutions (acidic, alkaline, or neutral), which were conducted using 0.05 M sulfuric acid solution, 0.1 M sodium hydroxide solution, or DI water, respectively. A liquid-to-biomass ratio (volume:mass) of 10:1 was used for all conditions, and the washes were performed at 80 °C for 3 h in a water bath. Acidic and alkaline preprocessed biomass were rinsed and soaked (50:1 water-to-biomass) with DI water at room temperature. Water was changed daily until the pH reached between 5 and 7. All preprocessed biomass were drained and centrifuged for 10 min, and the solids were stored at -20 °C until used. A portion of each WTC sample (including untreated) was manually separated into white wood (stem) and bark. Branches were peeled and separated.

The preprocessing conditions were chosen based on biomass washing techniques established previously by our research group [17] and on deacetylation studies from Chen et al. [37], which have obtained promising results on the removal of NSC from the biomass with low sugar loss.

All samples, including the fractions, were analyzed regarding sugars, lignin, total ash content, elemental composition, and total extractives.

### Steam explosion pretreatment

Steam explosion (SE) pretreatment was performed in duplicate for all samples. Briefly, 300 g of oven-dried (OD) biomass was impregnated with anhydrous 3% (w/w) SO2 overnight, and then steam exploded at 195 °C for 5 min in a 2.7 L batch reactor (Aurora Technical, Savona, BC, Canada). After SE, the pretreated biomass slurry was vacuum filtered using filter paper to separate the solid and liquid fractions. The solid fraction was washed using DI water, and the wash-water was analyzed by High-Pressure Liquid Chromatography (HPLC), as described below. The solids were washed until no residual soluble sugars and inhibitors originated from the liquid stream were detected.

### Compositional analysis

#### High-Pressure Liquid Chromatography (HPLC) analysis

Monomeric sugar concentration was determined using a Dionex HPLC ICS-3000 system (Sunnyvale, CA) equipped with an electrochemical detector and anion exchange column (Dionex, CarboPac PA1), using DI water at 1 mL/min flow rate as eluent and post-column addition of 0.2 M NaOH at 0.5 mL/min.

Acetic acid, furfural, 5-hydroxymethylfurfural (HMF), and ethanol concentrations were determined using refractive index detection (RID) on a Shimadzu Prominence LC equipped with an anion exchange column (Rezex RHM Monosaccharide H + (8%) Phenomenex, Inc., Torrance, CA), using mobile phase 5 mM H2SO4 at 0.6 mL/min flow rate.

#### Ash and extractives

The total ash content of all solid samples, including untreated and preprocessed biomass, as well as the solid fraction after SE, was measured gravimetrically according to a NREL procedure, by heating 0.5 g of OD 40 mesh-ground sample in a muffle furnace at 575 °C for 12 h [38]. Total organic extractives content (which is called “extractives” in this work for simplicity) of untreated and preprocessed biomass was determined according to NREL procedure [39], with a reflux time of 12 h for both water and ethanol Soxhlet extraction.

#### Elemental analysis

The specific mineral content of all solid samples was determined by elemental analysis. Briefly, 40 mesh-ground OD samples were digested in series with nitric acid, hydrogen peroxide, and hydrochloric acid at 155 °C for 5 h. The sample digest was filtered, and the filtrate was analyzed for mineral composition using inductively coupled plasma optical emission spectrometry (ICP-OES, Thermo-Scientific, iCAP 6300) [40].

#### Solid fraction carbohydrates, acetate groups, and acid-soluble lignin

The chemical composition of all solid samples was determined according to methods derived from TAPPI Standard Method T 222 [41] and NREL procedure [42]. Briefly, 0.2 g of 40 mesh-ground OD sample was treated with 3 mL of 72% H_2_SO_4_ for 2 h at room temperature, followed by dilution with 112 mL of DI water and then autoclaved at 121 °C for 60 min. Acid-insoluble lignin content was determined gravimetrically by filtration through tared sintered glass crucibles. Following filtration, the filtrate was analyzed by HPLC, as described previously (for sugars and acetyl content determination) and by UV/Vis spectrophotometer (Shimadzu UV-1800, Tokyo, Japan) at 205 nm for acid-soluble lignin content determination.

#### Liquid fraction carbohydrates and degradation products

Monomeric and oligomeric soluble carbohydrates present in the liquid fraction after SE were quantified based on the NREL procedure [43]. Briefly, 0.7 mL of 72% H_2_SO_4_ and 4.3 mL of DI water were added to 15 mL of the liquid sample. The mixture was autoclaved at 121 °C for 60 min and analyzed by HPLC, as described previously. Oligomeric sugars were calculated by subtracting monomeric sugar content from the total sugar content.

The concentration of furfural, 5-hydroxymethylfurfural (HMF), and acetic acid were determined by HPLC, as described previously. Total phenolic concentration was determined by the Folin-Ciocalteu method [44] using a UV/Vis spectrophotometer at 765 nm (Shimadzu UV-1800, Tokyo, Japan). Gallic acid was used as a calibration standard for total phenolics.

### Buffering capacity test

The buffering capacity of untreated and preprocessed biomass was determined by titration as described by Hӧrhammer et al. [17]. Briefly, 50 g OD weight of biomass was soaked in 1 L of DI water at 80 °C for 30 min in a water bath. Next, biomass was separated by vacuum filtration, and 800 mL of filtrate was titrated with 50 mL of 0.004 M H_2_SO_4_. DI water was titrated as blank.

### Enzymatic hydrolysis

Enzymatic hydrolysis of the solid fraction after SE was carried out at 5% (m/v) solids consistency in a total volume of 50 mL in an orbital shaker at 50 °C and 175 rpm. 50 mM sodium citrate buffer was used to maintain the pH at 4.8. Cellulase (Celluclast 1.5L, Sigma) was added at 67 mg protein/g cellulose to the pretreated biomass, supplemented with β-glucosidase (Novozyme 188, Sigma) at 9 mg protein/g cellulose. 1 mL samples were collected at multiple time points to quantify the cellulose to glucose conversion. All glucose-rich liquids were filtered after 96 h of reaction and boiled for 10 min. Finally, the liquid samples were stored at -20 °C until used for fermentation.

### Monomeric sugar yield and recovery calculation

The monomeric sugar yield and recovery after SE and EH were calculated based on the input feedstock mass and its original sugar composition, respectively. The output total monomeric sugar was obtained by combining the monomeric sugars in the liquid fraction after pretreatment with the monomeric glucose and xylose released after EH of the solid fraction. Monomeric sugar yield is equal to the total mass of monomeric sugars found in both solid and liquid fractions divided by the initial OD mass of raw biomass times 1000 (kg monomeric sugars/tonne of OD raw biomass). Monomeric sugar recovery, on the other hand, is defined as the total mass of monomeric sugars found in both solid and liquid fractions divided by the initial monomeric sugar mass in the raw biomass (kg monomeric sugars recovered/kg original monomeric sugars × 100%).

### Overliming

Poplar CPC liquid fractions after steam pretreatment were conditioned by overliming based on the procedure described by Mohagheghi et al. [12] with modifications. Briefly, the liquid fraction’s pH was increased to 10 with Ca(OH)_2_ (calcium hydroxide) and incubated at 50 °C for 1 h with rotation at 150 rpm. Next, the liquid was filtered through a 0.2 µm filter (Fisherbrand disposable PES filter) and the filtrate pH was readjusted to 6 by adding 10 N H_2_SO_4_ solution. The filtered liquid was incubated at 50 °C for 1 h with rotation at 150 rpm, followed by a final sterile filtration (0.2 µm Fisherbrand disposable PES filter).

### Fermentation

#### Microorganism and media

*Scheffersomyces stipitis* ATCC 58376, also known as *Pichia stipitis* Y-7124, was obtained from ATCC, Manassas, VA. Single colonies were transferred from agar plates to a sterile medium containing 10 g/L glucose, 20 g/L xylose, 3 g/L yeast extract, 5 g/L peptone, 2.3 g/L urea, and 1 g/L MgSO_4_·7H_2_O. The inoculum was grown at 30 °C with constant orbital mixing (175 rpm) for 48 h. Cells were harvested by centrifugation at 1500 g-force for 5 min at room temperature. The pellets were washed and resuspended in sterile DI water to obtain a concentrated yeast culture. Cell concentration was measured using a UV/Vis spectrophotometer (Shimadzu UV-1800) based on a standard curve relating the dry cell weight (DCW) per liter with its corresponding absorbance at 600 nm.

#### Fermentation

Fermentation was performed separately on solid fraction after EH and liquid fraction after pretreatment (with and without overliming). Specifically for liquid fraction fermentation, one sample of each type (acidic, alkaline, and neutral) was randomly selected among its replicates. All samples were supplemented with 3 g/L yeast extract, 5 g/L peptone, 2.3 g/L urea, and 1 g/L MgSO_4_·7H_2_O, and the pH was brought to 6 using a 50% NaOH solution. All samples were filter-sterilized (0.2 µm Fisherbrand disposable PES filter) before inoculation. A control was prepared using reagent-grade sugars at similar concentrations to those in the experimental samples. Fermentation was performed in duplicate using 125 mL foam-plugged Erlenmeyer flasks (semi-aerobic) at 30 °C and 175 rpm, with 50 mL total volume. Concentrated yeast culture was added to achieve 5 g of DCW per liter. 1 mL samples were aseptically collected at the time of inoculation and at multiple time points. They were immediately centrifuged at 9600 g-force for 5 min at room temperature, and the cell-free supernatant was analyzed by HPLC for sugar and ethanol quantification, as described previously.

#### Ethanol yield and percent theoretical yield calculations

The ethanol yield was calculated based on the ratio between the maximum ethanol concentration achieved during fermentation and the total initial sugar concentration. A theoretical maximum ethanol yield of 0.51 per unit of sugar (g/g) was used to calculate the percent theoretical yield (Y _%T_) [17] (Eq. 1).

Equation 1. Percent theoretical ethanol yield.

1 $${\text{Y}}_{{{\text{\% T}}}} = \frac{\text{maximum ethanol concentration}}{{{\text{initial sugars concentration }} \times 0.51}} \times 100$$

### Statistical analysis

Biomass chemical composition and total monomeric sugars yield and recovery were subjected to one-way analysis of variance (ANOVA) followed by a Tukey’s test based on a 5% alpha level (95% confidence interval). Data were analyzed using Minitab 18 software. Each experiment was analyzed in triplicate unless otherwise stated. Any data analysis mentioned as “significant” represents statistically significant (*p* < 0.05).

### Economic assessment

#### Incremental Return on Investment (ROI)

The incremental return on investment (ROI) associated with making changes in the ethanol production process was calculated as shown in Eq. 2. The ROI comprises of pairwise comparison between different scenarios (A and B) by considering the additional revenue and capital cost associated with the process changes, and it indicates how much of the initial investment is recovered annually with the proposed changes.

Equation 2. Incremental Return on Investment (ROI) calculation.

2 $$\begin{aligned} {\text{Incremental ROI }}\left( {\text{\%}} \right) & = \frac{\text{additional revenue}}{\text{additional capital cost}} \times 100 \\ & = \frac{{({\text{revenue}} - {\text{operating costs}})_{\text{A}} - ({\text{revenue}} - {\text{operating costs}})_{\text{B}} }}{{\left( {\text{capital cost}} \right)_{\text{A}} - \left( {\text{capital cost}} \right)_{\text{B}} }} \times 100 \\ \end{aligned}$$

## Supplementary information

**Additional file 1.** Table S1 Chemical composition of white wood and bark fractions of WTC poplar samples; Table S2 Elemental composition of white wood and bark fractions of WTC samples; Table S3 Yields of sugars, lignin, and ash (kg/tonne) of solid fraction after steam pretreatment; Table S4 Monomeric sugar yield and recovery after steam pretreatment and enzymatic hydrolysis; Table S5 Initial concentration of sugars (glucose and xylose) and maximum ethanol concentration during fermentation; Table S6 Individual equipment prices (based on NREL reports and adjusted to in-house size); Table 7 Individual price quotations used for calculating operating costs.

## Data Availability

All data generated or analyzed during this study are included in this article and its additional files.

## Abbreviations

NSC: Non-structural components
SRC: Short rotation coppice
CPC: Clean pulp chips
WTC: Whole-tree chips
WWT: Wastewater treatment
MM: One million
FPU: Filter paper units
HPLC: High-pressure liquid chromatography
OD: Oven-dried
DI: Deionized
HMF: 5-Hydroxymethyl furfural
DCW: Dry cell weight
EH: Enzymatic hydrolysis
SE: Steam explosion
NREL: National Renewable Energy Laboratory
ROI: Return on investment
