# Supplementary material for: Novel ethanol production using biomass preprocessing to increase ethanol yield and reduce overall costs
Source: Biotechnol Biofuels. 2021 Jan 7;14:9. doi: 10.1186/s13068-020-01839-0 (PMC7789555; doi:10.1186/s13068-020-01839-0)
Supplement: Supplementary file 1 — Additional file 1. Table S1 Chemical composition of white wood and bark fractions of WTC poplar samples; Table S2 Elemental composition of white wood and bark fractions of WTC samples; Table S3 Yields of sugars, lignin, and ash (kg/tonne) of solid fraction after steam pretreatment; Table S4 Monomeric sugar yield and recovery after steam pretreatment and enzymatic hydrolysis; Table S5 Initial concentration of sugars (glucose and xylose) and maximum ethanol concentration during fermentation; Table S6 Individual equipment prices (based on NREL reports and adjusted to in-house size); Table 7 Individual price quotations used for calculating operating costs. [file 13068_2020_1839_MOESM1_ESM.docx]

## Additional material

**Table S.** Chemical composition of white wood and bark fractions of WTC poplar samples.

|  |  | **Glucan (%)** | **Xylan (%)** | **Total sugars* (%)** | **Total lignin (%)** | **Acetic acid (%)** | **Total ash (%)** | **Total extractives (%)** |
| --- | --- | --- | --- | --- | --- | --- | --- | --- |
| **Untreated** | **White wood** | 45.0 ± 0.8 | 18.0 ± 0.1 | 65.8 ± 0.9 | 28.5 ± 1.6 | 5.5 ± 0.1 | 0.5 ± 0.0 | 6.3 ± 0.1 |
|  | **Bark** | 33.2 ± 0.6 | 6.8 ± 0.1 | 46.0 ± 0.8 | 33.8 ± 1.2 | 2.0 ± 0.0 | 6.5 ± 0.0 | 28.2 ± 0.7 |
| **Acidic** | **White wood** | 47.8± 0.4 | 19.1 ± 0.1 | 69.4 ± 0.5 | 29.6 ± 0.8 | 5.6 ± 0.2 | 0.2 ± 0.1 | 4.2 ± 0.0 |
|  | **Bark** | 32.5 ± 0.6 | 8.9 ± 0.4 | 45.5 ± 1.2 | 34.4 ± 1.6 | 2.6 ± 0.2 | 6.1 ± 0.2 | 13.2 ± 0.1 |
| **Alkaline** | **White wood** | 49.1 ± 0.5 | 19.6 ± 0.2 | 71.6 ± 0.7 | 28.0 ± 0.8 | 4.3 ± 0.0 | 0.5 ± 0.1 | 3.6 ± 0.1 |
|  | **Bark** | 34.5 ± 0.6 | 9.4 ± 0.2 | 51.6 ± 0.9 | 30.6 ± 0.5 | 1.1 ± 0.0 | 7.8 ± 0.2 | 7.4 ± 0.5 |
| **Neutral** | **White wood** | 46.3 ± 1.7 | 18.1 ± 0.4 | 67.1 ± 2.2 | 26.9 ± 0.6 | 5.8 ± 0.4 | 0.5 ± 0.0 | 5.2 ± 0.3 |
|  | **Bark** | 31.0 ± 1.9 | 7.6 ± 0.6 | 46.1 ± 3.0 | 27.9 ± 1.2 | 2.5 ± 0.0 | 7.6 ± 0.0 | 13.6 ± 0.3 |

Data represented as the mean values of triplicate analysis with standard deviation, extractives as duplicates.

*Total sugars include glucan, xylan, arabinan, galactan, and mannan.

**Table S.** Elemental composition of white wood and bark fractions of WTC samples.

|  |  | **Ba (µg/g)** | **Ca (µg/g)** | **K (µg/g)** | **Mg (µg/g)** | **Na (µg/g)** | **P (µg/g)** | **S (µg/g)** | **Si (µg/g)** |
| --- | --- | --- | --- | --- | --- | --- | --- | --- | --- |
| **Untreated** | **White wood** | 8 ± 0.1 | 1234 ± 30 | 1143 ± 18 | 292 ± 38 | 0.0 ± 0.0 | 426 ± 38 | 143 ± 1 | 43 ± 1 |
|  | **Bark** | 87 ± 4 | 13145 ± 171 | 6723 ± 674 | 1187 ± 106 | 0.0 ± 0.0 | 1137 ± 15 | 622 ± 29 | 48 ± 2 |
| **Acidic** | **White wood** | 4 ± 0.2 | 239 ± 13 | 0.0 ± 0.0 | 0.0 ± 0.0 | 0.0 ± 0.0 | 118 ± 0.7 | 78 ± 2 | 0.0 ± 0.0 |
|  | **Bark** | 87 ± 1 | 16455 ± 279 | 0.0 ± 0.0 | 212 ± 4 | 0.0 ± 0.0 | 993 ± 35 | 687 ± 12 | 32 ± 1 |
| **Alkaline** | **White wood** | 8 ± 0.0 | 917 ± 22 | 0.0 ± 0.0 | 220 ± 16 | 532 ± 0.0 | 178 ± 11 | 75 ± 5 | 32 ± 2 |
|  | **Bark** | 127 ± 2 | 21180 ± 430 | 298 ± 4 | 1103 ± 84 | 1430 ± 25 | 772 ± 61 | 505 ± 20 | 24 ± 1 |
| **Neutral** | **White wood** | 7 ± 0.1 | 872 ± 19 | 461 ± 15 | 229 ± 10 | 0.0 ± 0.0 | 334 ± 9 | 112 ± 1 | 28 ± 1 |
|  | **Bark** | 98 ± 3 | 17580 ± 425 | 2568 ± 111 | 1203 ± 58 | 0.0 ± 0.0 | 1008 ± 3 | 585 ± 17 | 47 ± 1 |

Data represented as the mean values of duplicate analysis with standard deviation.

**Table S.** Yields of sugars, lignin, and ash (kg/tonne) of solid fraction after steam pretreatment.

|  |  | **Glucan** | **Xylan** | **Lignin** | **Ash** |
| --- | --- | --- | --- | --- | --- |
|  |  | **kg/tonne** | **kg/tonne** | **kg/tonne** | **kg/tonne** |
| **Whole-tree chips (WTC)** | **Untreated** | 384 ± 32 | 15 ± 1 | 232 ± 19 | 6 ± 0 |
|  | **Acidic** | 395 ± 12 | 8 ± 1 | 242 ± 2 | 1 ± 0 |
|  | **Alkaline** | 427 ± 4 | 18 ± 0 | 238 ± 5 | 4 ± 0 |
|  | **Neutral** | 388 ± 1 | 10 ± 1 | 236 ± 1 | 6 ± 0 |
| **Clean pulp chips (CPC)** | **Untreated** | 434 ± 2 | 7 ± 0 | 220 ± 5 | < 0 |
|  | **Acidic** | 425 ± 12 | 0 ± 0 | 214 ± 3 | < 0 |
|  | **Alkaline** | 527 ± 2 | 17 ± 0 | 204 ± 4 | < 0 |
|  | **Neutral** | 484 ± 11 | 10 ± 1 | 216 ± 0 | < 0 |

**Table S.** Monomeric sugar yield and recovery after steam pretreatment and enzymatic hydrolysis.

|  | **Total monomeric sugar recovery (%)** | |  | **Total monomeric sugar yield (kg/tonne)** | |
| --- | --- | --- | --- | --- | --- |
|  | **Whole-tree chips** | **Clean pulp chips** |  | **Whole-tree chips** | **Clean pulp chips** |
| **Untreated** | 73.2 ± 2.4^a^ | 75.0 ± 0.9^ac^ |  | 493.0 ± 16.0^a^ | 539.1 ± 6.1^a^ |
| **Acidic** | 87.5 ± 1.2^b^ | 80.0 ± 0.6^b^ |  | 578.3 ± 8.2^b^ | 577.5 ± 4.4^b^ |
| **Alkaline** | 80.6 ± 0.1^ab^ | 72.9 ± 1.4^c^ |  | 553.0 ± 0.7^ab^ | 573.1 ± 10.7^b^ |
| **Neutral** | 82.3 ± 5.8^ab^ | 78.1 ± 0.2^ab^ |  | 528.7 ± 37.6^ab^ | 579.7 ± 1.7^b^ |

Different superscript letters indicate statistically significant differences (p<0.05) within each column by Tukey’s test (WTC and CPC treatments were compared separately).

**Table S.** Initial concentration of sugars (glucose and xylose) and maximum ethanol concentration during fermentation.

|  |  | **Solid fraction after EH** | | | **Liquid fraction** | | | **Overlimed liquid fraction** | | |
| --- | --- | --- | --- | --- | --- | --- | --- | --- | --- | --- |
|  |  | **Glucose^1^ (g/L)** | **Xylose^1^ (g/L)** | **Ethanol^2^ (g/L)** | **Glucose^1^ (g/L)** | **Xylose^1^ (g/L)** | **Ethanol^2^ (g/L)** | **Glucose^1^ (g/L)** | **Xylose^1^ (g/L)** | **Ethanol^2^ (g/L)** |
|  | **Control ^3^** | 28.3 ± 0.3 | 0.0 ± 0.0 | 12.2 ± 0.7 | 7.1 ± 0.4 | 27.1 ± 0.3 | 13.8 ± 0.0 | 7.8 ± 0.0 | 23.0 ± 0.1 | 12.2 ± 0.1 |
| **Whole-tree chips (WTC)** | **Untreated** | 31.6 ± 4.0 | 1.3 ± 0.1 | 13.8 ± 1.7 | 18.8 ± 0.0 | 44.8 ± 0.0 | 0.6 ± 0.1 | NA | NA | NA |
|  | **Acidic** | 32.8 ± 0.7 | 0.9 ± 0.1 | 14.3 ± 0.4 | 13.4 ± 0.0 | 37.2 ± 0.1 | 13.7 ± 0.3 | NA | NA | NA |
|  | **Alkaline** | 31.1 ± 1.9 | 1.4 ± 0.1 | 13.6 ± 0.9 | 7.6 ± 0.1 | 30.0 ± 0.2 | 10.5 ± 0.0 | NA | NA | NA |
|  | **Neutral** | 31.3 ± 0.7 | 0.9 ± 0.1 | 13.7 ± 0.4 | 12.3 ± 0.0 | 35.6 ± 0.1 | 12.0 ± 0.1 | NA | NA | NA |
| **Clean pulp chips (CPC)** | **Untreated** | 37.4 ± 0.7 | 0.9 ± 0.0 | 16.4 ± 0.4 | 17.6 ± 0.1 | 44.1 ± 0.1 | 0.4 ± 0.0 | 19.1 ± 0.4 | 28.7 ± 0.4 | 11.0 ± 0.1 |
|  | **Acidic** | 39.0 ± 1.3 | 0.7 ± 0.1 | 16.7 ± 0.6 | 20.4 ± 0.2 | 34.4 ± 0.0 | 14.5 ± 0.8 | 18.7 ± 0.7 | 26.0 ± 0.4 | 12.2 ± 0.1 |
|  | **Alkaline** | 38.3 ± 1.5 | 1.8 ± 0.2 | 16.7 ± 0.7 | 7.0 ± 0.1 | 32.3 ± 0.1 | 11.0 ± 0.1 | 8.0 ± 0.2 | 26.4 ± 0.3 | 11.3 ± 0.1 |
|  | **Neutral** | 38.1 ± 1.1 | 0.9 ± 0.1 | 17.0 ± 0.0 | 12.5 ± 0.1 | 32.5 ± 0.4 | 12.8 ± 0.3 | 12.9 ± 0.1 | 24.5 ± 0.3 | 10.4 ± 0.1 |

Data represented as the mean values of duplicate analysis with standard deviation

NA = not applicable

^1^ Glucose and xylose concentration at the beginning of the fermentation (time 0h)

^2^ Maximum ethanol concentration

^3^ The fermentation control contained reagent-grade sugars at similar concentrations to those in experimental samples with lower overall sugar concentration

**Table S.** Individual equipment prices (based on NREL reports and adjusted to in-house size).

| **Equipment Name** | **Installed cost in 2018$ (in-house size)** |
| --- | --- |
| Overliming Tank Agitator | $ 36,658.50 |
| Reacidification Tank Agitator | $ 111,597.94 |
| Lime Solids Feeder | $ 5,510.73 |
| Overlimed Hydrolyzate Pump | $ 98,986.00 |
| Filtered Hydrolyzate Pump | $ 100,574.58 |
| Lime Unloading Blower | $ 151,751.76 |
| Reacidified Liquor Pump | $ 100,147.00 |
| Hydroclone & Rotary Drum Filter | $ 285,243.22 |
| LimeDust Vent Baghouse | $ 233,596.55 |
| Overliming Tank | $ 157,390.36 |
| Lime Storage Bin | $ 194,301.79 |
| Reacidification Tank | $ 252,978.15 |
| **OVERLIMING TOTAL** | **$ 1,728,736.58** |
|  |  |
| Preprocessing reactor | $ 2,938,248.79 |
| Preprocessing reactor conveyors | $ 414,578.25 |
| **PREPROCESSING TOTAL** | **$ 3,352,827.03** |

**Table S.** Individual price quotations used for calculating operating costs.

| **Description** | **Cost 2019 ($/tonne)** | **Quote source** |
| --- | --- | --- |
| CPC chips (dry) | $ 116 | Greenwood Resources |
| WTC chips (dry) | $ 77 | Greenwood Resources |
| Calcium hydroxide | $ 165 | Kemcore |
| Sulfuric acid | $ 41 | Echemi.com |
| Gypsum disposal | $ 55 | Waste Business Journal |
| Fresh water | $ 5 | EPA 2017 Report |
